# New Solutions for “Old” Problems: Implications and Opportunities of Intergenerational HomeSharing

**DOI:** 10.1093/geroni/igab046.2871

**Published:** 2021-12-17

**Authors:** Raza Mirza, Jacalyn Tanner, James Hull, Taylor Hocking, Anna Liu, Jessica Hsieh, Christopher Klinger

**Affiliations:** 1 University of Toronto, Toronto, Ontario, Canada; 2 National Initiative for the Care of the Elderly (NICE), Toronto, Ontario, Canada

## Abstract

Across North America, many older adults have expressed their preference to live in their own homes and communities for as long as possible — and to 'age in place'. To address challenges faced by older adults living in the community, home-sharing - an exchange-based intergenerational housing approach, has empowered older adults to ‘thrive in place’ by providing additional income, companionship, and support with household tasks. In 2018, Toronto HomeShare was launched as an intergenerational home-sharing pilot program (n=22), matching older adults (55+) with postsecondary students intending to simultaneously address social isolation and the affordable housing crisis. In 2019, the pilot was adopted as a funded program in the City of Toronto with over 200 participants. Program results highlight unique benefits and challenges for older adults participating in home-sharing: (1) the capacity for intergenerational engagement to fulfill social needs, and (2) the importance of agency facilitation as a determinant of the experience for older adults. Survey findings indicate 88% of participants reported that participation in HomeShare positively impacted their general well-being, 88% reported improved financial security, 94% reported a delay in the need to move out of their community, and 72% felt that participation in HomeShare prevented the need for institutional care. These findings were used to transition Toronto HomeShare into a fully funded program as well as in the development of a national program. Beginning in January 2021 Toronto HomeShare transitioned to Canada HomeShare and will be scaling the program to Vancouver, Winnipeg, Halifax, Calgary, Montreal and other Canadian cities.

